# Development and validation of an interpretable machine learning model for predicting atrial fibrillation risk in middle-aged and older patients with coronary heart disease

**DOI:** 10.3389/fcvm.2026.1886992

**Published:** 2026-07-08

**Authors:** Feng Chen, Qin Fu, Ling Li, Xiao Zhang, Yongqiong Ge, Jianfei Chen

**Affiliations:** 1 Department of Cardiology, Affiliated Banan Hospital of Chongqing Medical University, Chongqing, China; 2 Department of Medical Records and Statistics, Affiliated Banan Hospital of Chongqing Medical University, Chongqing, China; 3 Department of Nursing, Affiliated Banan Hospital of Chongqing Medical University, Chongqing, China

**Keywords:** atrial fibrillation, coronary heart disease, electronic medical record, Shapley additive explanations, XGBoost

## Abstract

**Background:**

Coronary heart disease (CHD) and atrial fibrillation (AF) frequently coexist, yet existing risk stratification tools inadequately capture the nonlinear, multidimensional determinants of AF in middle-aged and older CHD patients. This study aimed to develop and validate an interpretable machine learning-based prediction model leveraging electronic medical records (EMR) data.

**Methods:**

A retrospective cohort of 47,617 hospitalized CHD patients (January 2020–December 2025) was analyzed. After random forest imputation and least absolute shrinkage and selection operator (LASSO) screening, eight machine learning algorithms were trained and validated (7:3 split). Model performance was evaluated by the area under the receiver operating characteristic curve (AUC), calibration curves, and decision curve analysis (DCA), with Shapley additive explanations (SHAP) and local interpretable model-agnostic explanations (LIME) applied for interpretability.

**Results:**

LASSO regression identified 16 predictors, with pulse rate, total cholesterol, systolic blood pressure, creatinine, and triglycerides emerging as the top five contributors. XGBoost outperformed competing models, achieving AUCs of 0.867 (95% CI: 0.862–0.872) in the training set and 0.813 (95% CI: 0.802–0.823) in the validation set. Restricted cubic spline analysis revealed nonlinear dose–response relationships for multiple continuous variables. SHAP visualization quantified individualized feature contributions. LIME explanations demonstrated consistent local feature contributions at the individual level.

**Conclusions:**

This data-driven, interpretable XGBoost model enables individualized AF risk assessment in middle-aged and older CHD patients, offering a practical tool for early identification and targeted intervention in clinical practice.

## Introduction

According to World Health Organization statistics, cardiovascular diseases (CVDs) represent the leading cause of death globally, claiming more lives annually than any other disease category, thereby posing a major public health challenge worldwide (1, 2). CVDs encompass a broad spectrum of disorders affecting the heart and vascular system, among which coronary heart disease (CHD) and atrial fibrillation (AF) are two common and highly detrimental types that have drawn considerable attention (3, 4). AF is the most frequently encountered cardiac arrhythmia in clinical practice, with an estimated prevalence of 2%–4% in the adult population; its incidence is expected to rise further in the coming years (5). AF is a major contributor to declining cardiac function and adverse cardio-cerebrovascular events such as thromboembolism, and its clinical impact should not be underestimated (6). CHD, the most prevalent form of CVD, represents a leading cause of death in both developed and developing countries. Globally, its age-standardized mortality rate increased from 99 per 100,000 population in 1990 to 116.4 per 100,000 in 2019 (7). Given current lifestyle trends, the incidence of CHD is projected to continue rising. Accumulating evidence indicates that patients with CHD are prone to developing AF, which further complicates treatment and leads to poor prognosis (8, 9).

Although existing evidence has clearly demonstrated that patients with CHD are at significantly increased risk of developing AF, and that the coexistence of both conditions leads to further deterioration of clinical outcomes, effective prediction tools specifically for AF risk in the CHD population remain relatively scarce (10, 11). Traditional risk assessment models, largely based on parametric approaches such as logistic regression, are insufficient for capturing the complex nonlinear relationships and interactions among variables (12). Moreover, most existing models have been developed in populations with isolated AF or in general community samples, with few prediction tools specifically tailored to middle-aged and older CHD patients (13, 14). Furthermore, these tools often fail to fully leverage the rich structured data available in electronic medical records (EMRs). Consequently, there is an urgent clinical need for a data-driven, interpretable AF risk prediction model tailored to middle-aged and older CHD patients, to facilitate early identification of high-risk individuals and guide personalized intervention strategies.

In recent years, machine learning (ML) algorithms have been extensively validated across a broad spectrum of clinical applications, largely owing to their exceptional capacity to model complex nonlinear dependencies and interactive effects among predictors (15, 16). This capability has been particularly well demonstrated in the domain of CVD risk assessment. Motivated by these advances, the present study seeks to develop a ML-based risk prediction tool specifically tailored to middle-aged and older patients with CHD. By integrating multidimensional clinical data, this tool aims to enable individualized assessment of AF risk. The establishment of such a model is expected to provide objective, evidence-based support for the early identification of high-risk individuals in clinical practice and the formulation of targeted intervention strategies.

## Methods

### Study design and participants

This study adopted a retrospective, data-driven design using anonymized EMRs collected from the Affiliated Banan Hospital of Chongqing Medical University. Hospitalized patients diagnosed with CHD between January 2020 and December 2025 were considered for enrollment. All data were extracted from the EMR system at the time of each patient's index hospitalization, as this was a retrospective cross-sectional study requiring no longitudinal follow-up. Exclusion criteria were as follows: (1) age <45 years; (2) hospital stay <24 h; (3) concurrent severe valvular heart disease, defined as moderate-to-severe mitral or aortic stenosis/regurgitation; and (4) concomitant malignancy. The initial cohort comprised 55,650 patients, and a final set of 47,617 eligible patients was included in the analysis. All eligible patients meeting the inclusion criteria during the study period were consecutively enrolled without any additional selection, ensuring representativeness of the target population. The dataset was subsequently divided into a training set (*n* = 33,331) and a validation set (*n* = 14,286) according to a 7:3 ratio. The patient selection and data partitioning workflow are illustrated in Supplementary Figure 1.

This study was approved by the Ethics Committee of the Affiliated Banan Hospital of Chongqing Medical University (approval number: BNLL-KY-2026-003). Owing to the exclusive use of anonymized historical data without any patient intervention, the committee waived the requirement for informed consent. The reporting of the prediction model adheres to the Transparent Reporting of a Multivariable Prediction Model for Individual Prognosis or Diagnosis (TRIPOD) and the TRIPOD-Artificial Intelligence (TRIPOD-AI) guidelines (17).

### Data collection and clinical definitions

In this study, CHD was defined based on formal clinician diagnoses recorded in the EMR system, following the International Classification of Diseases, Tenth Revision (ICD-10) criteria. This study adopted a cross-sectional design. The primary outcome was the presence of AF during the index hospitalization, defined by ICD-10 criteria and confirmed by at least one 12-lead electrocardiogram (ECG) report or continuous ECG monitoring record during the same hospitalization. All diagnoses were made by attending cardiologists, and a second independent physician reviewed a random 5% sample of cases to verify diagnostic accuracy. Thus, the outcome represents prevalent AF rather than incident AF after discharge. No longitudinal follow-up was performed, and no time-to-event data were collected. The extracted variables covered demographic characteristics, comorbidities, vital signs, and laboratory parameters, specifically including age, sex, marital status, smoking history, drinking history, surgical history, heart failure (HF), diabetes mellitus (DM), hypertension, chronic obstructive pulmonary disease (COPD), chronic gastritis (CG), hypokalemia, pulse rate, systolic blood pressure (SBP), diastolic blood pressure (DBP), total cholesterol (TC), triglycerides (TG), creatinine (CREA), uric acid (UA), potassium (K), phosphorus (P), chloride (Cl), sodium (Na), and calcium (Ca). Additionally, to explore their potential predictive value, ten novel composite indicators were incorporated: neutrophil-to-lymphocyte ratio (NLR), platelet-to-lymphocyte ratio (PLR), lymphocyte-to-monocyte ratio (LMR), pan-immune-inflammation value (PIV), fibrosis-4 index (FIB-4), prognostic nutritional index (PNI), fibrinogen-to-albumin ratio (FAR), low-density lipoprotein cholesterol/high-density lipoprotein cholesterol (LDL-C/HDL-C), non-high-density lipoprotein cholesterol to high-density lipoprotein cholesterol ratio (NHHR), and c-reactive protein/albumin (CRP/ALB).

### Data pre-processing

During preprocessing, features with more than 30% missing data were removed, while those with missing rates up to 30% were imputed using the random forest algorithm (18). Missing proportions for each variable in both the training and validation sets are presented in Supplementary Tables 1, 2, respectively. The ten novel composite indicators were calculated after imputation using their respective component variables. No data augmentation techniques were employed, as this was a cross-sectional study with structured tabular data. The imputation procedure consisted of the following steps. For each feature containing missing values, a random forest regression model was constructed using the remaining features as inputs. Initial missing values were first filled with simple statistics such as the mean or median. The model was then trained on complete cases (samples without any missing values) and subsequently used to predict the missing entries, replacing the initially imputed values. This process was repeated iteratively until model convergence or a preset number of iterations was reached, ensuring stable imputation outcomes.

### Model development and validation

To minimize overfitting and identify the optimal set of predictors, least absolute shrinkage and selection operator (LASSO) regression was applied to reduce the dimensionality of all variables showing statistically significant intergroup differences (19). Because the outcome is prevalent AF (a binary status) in a cross-sectional design without time-to-event data, binary classification ML models are methodologically appropriate and were therefore employed in this study. A total of eight distinct ML models were subsequently developed and evaluated: extreme gradient boosting (XGBoost), gradient boosting decision tree (GBDT), light gradient boosting machine (LightGBM), random forest (RF), adaptive boosting (AdaBoost), logistic regression (LR), decision tree (DT), and naive Bayes (NB). Hyperparameter optimization for all models was performed using grid search with 5-fold cross-validation on the training set; the final hyperparameters are summarized in Supplementary Table 3. All models were implemented using Python (version 3.12.3) with the scikit-learn (1.1.3), xgboost (1.6.2), and lightgbm (3.3.2) libraries, and were trained on a workstation equipped with an Intel Core i7-12700K CPU (3.6 GHz, 12 cores) and 64 GB RAM, without GPU acceleration. For XGBoost, the loss function was binary logistic (binary: logistic) with AUC as the evaluation metric, and early stopping with 50 rounds of patience was applied to prevent overfitting. The scale_pos_weight parameter was set to the imbalance ratio (non-AF/AF) to account for class imbalance.

Each model was subjected to training, prediction, and performance evaluation. To ensure robust performance estimation, we performed stratified 5-fold cross-validation on the training set for each algorithm, with stratification based on AF status to preserve the class distribution across folds. Given the inherent class imbalance in AF prediction, we did not employ oversampling techniques such as SMOTE or synthetic data augmentation, as these could introduce artificial biases and distort the real-world clinical distribution. Instead, we relied on: (1) stratified cross-validation to maintain consistent class proportions across folds; and (2) comprehensive per-class performance reporting to transparently evaluate model performance on both AF and non-AF cases. This approach preserves the natural disease prevalence while allowing rigorous assessment of minority-class prediction. The mean AUC and standard deviation (SD) across the five folds were calculated to assess model stability (20). Additionally, the final model was evaluated on the hold-out validation set (7:3 random split). Key metrics, including the area under the receiver operating characteristic curve (AUC), sensitivity, specificity, Youden index, and F1 score, along with their 95% confidence intervals (CIs), were collected and analyzed. For each model, we also report per-class performance metrics (precision, recall, and F1-score) for both the AF and non-AF groups to provide a detailed breakdown of classification performance. Calibration curves were constructed to assess the agreement between predicted probabilities and the observed prevalence of AF. In addition, decision curve analysis (DCA) was performed to quantify the net benefit of each model across a range of threshold probabilities, based on the trade-off between anticipated gains and potential risks associated with the predictions.

Furthermore, to facilitate a more interpretable presentation of model outputs, Shapley additive explanations (SHAP) visualization was performed (21). This analysis focused primarily on the best-performing model in terms of predictive accuracy, quantifying the individual contribution of each variable. Specifically, SHAP values reflect the marginal contribution of a given feature to the predicted outcome: positive values indicate that the feature increases the likelihood of a positive outcome, whereas negative values suggest a reduced likelihood of such an outcome. To provide complementary interpretability, we additionally applied Local Interpretable Model-agnostic Explanations (LIME) to the same XGBoost model. While SHAP provides global and locally consistent feature importance based on game theory, LIME offers local decision-boundary explanations by approximating the model's behavior in the vicinity of each individual prediction (22). The combined use of SHAP and LIME enables both global feature ranking and case-level decision understanding.

### Ablation analysis

To systematically evaluate the contribution of individual model components, we performed three sets of ablation experiments on the validation set using the best-performing model identified from the eight candidate algorithms: (1) feature ablation, by sequentially removing the top-five SHAP-ranked predictors from the full feature set and retraining the model at each step; (2) preprocessing ablation, by comparing random forest imputation against mean/median imputation and complete-case analysis; and (3) optimizer ablation, by comparing grid search-optimized hyperparameters against default parameters. All ablations were evaluated using the validation set AUC to quantify the marginal contribution of each component.

### Statistical analysis

Because the data did not follow a normal distribution, continuous variables were summarized as medians with interquartile ranges, and between-group comparisons were performed using the Mann–Whitney *U* test. Categorical variables were presented as counts (percentages) and analyzed using the chi-square test. For variables selected by LASSO regression, collinearity was further assessed by calculating the variance inflation factor (VIF) and its reciprocal (tolerance). A correlation heatmap was constructed and correlation coefficients were computed for the quantitative variables included, in order to evaluate the strength of associations among them. To explore the dose-response relationship between continuous variables (e.g., pulse rate, SBP, DBP) and the risk of AF, restricted cubic spline (RCS) regression with three to five knots was applied (23). The optimal number of knots was determined based on the Akaike information criterion (AIC). Nonlinearity was formally tested, and the corresponding *P*-values for nonlinearity are reported. All statistical analyses were conducted with R software (version 4.3.3). A two-sided *P*-value <0.05 was considered statistically significant. Pairwise comparisons of AUCs between the best-performing model and each of the other seven models were performed using the Delong test on the validation set, with Benjamini–Hochberg false discovery rate (FDR) correction for multiple comparisons. All analytical code, including scripts for data preprocessing, model training, hyperparameter optimization, evaluation, and SHAP visualization, has been made publicly available at https://github.com/yoosl/CF.

## Results

### Baseline clinical characteristics of the enrolled population

The final analytic cohort comprised 47,617 patients, all of whom were randomly assigned in a 7:3 ratio to either a training cohort (*n* = 33,331) or a validation cohort (*n* = 14,286). Baseline characteristics of the two groups are summarized in Table 1. The median age of the cohort was 74 years, with males accounting for 51.66% of the population, and the majority of patients were married (89.03%). The proportions of patients with smoking history, drinking history, and surgical history were 30.98%, 12.73%, and 25.54%, respectively. Hypertension was the most common comorbidity (67.57%), followed by HF (52.04%) and DM (35.89%). The imputation results for missing variables in the training and validation sets are presented in Supplementary Tables 4, 5, respectively. Most variables showed no statistically significant differences before and after imputation (*P* > 0.05), indicating satisfactory imputation performance.

**Table 1:** Baseline characteristics of participants in the training and validation sets.

Variables	Total (*n* = 47,617)	Training set (*n* = 33,331)	Validation set (*n* = 14,286)	*P* value
Age (years)	74.00 (67.00, 81.00)	74.00 (66.00, 81.00)	74.00 (67.00, 81.00)	0.876
Sex				0.523
male	24,597 (51.66)	17,185 (51.56)	7,412 (51.88)	
female	23,020 (48.34)	16,146 (48.44)	6,874 (48.12)	
Marital status				0.955
married	42,395 (89.03)	29,684 (89.06)	12,711 (88.98)	
unmarried	2,279 (4.79)	1,594 (4.78)	685 (4.79)	
divorced/widowed	2,943 (6.18)	2,053 (6.16)	890 (6.23)	
Smoking history				0.187
No	32,863 (69.02)	23,065 (69.20)	9,798 (68.58)	
Yes	14,754 (30.98)	10,266 (30.80)	4,488 (31.42)	
Drinking history				0.559
No	41,554 (87.27)	29,107 (87.33)	12,447 (87.13)	
Yes	6,063 (12.73)	4,224 (12.67)	1,839 (12.87)	
Surgical history				0.417
No	35,454 (74.46)	24,853 (74.56)	10,601 (74.21)	
Yes	12,163 (25.54)	8,478 (25.44)	3,685 (25.79)	
HF				0.222
No	22,835 (47.96)	14,809 (44.43)	8,026 (56.18)	
Yes	24,782 (52.04)	18,522 (55.57)	6,260 (43.82)	
DM				0.062
No	30,528 (64.11)	21,459 (64.38)	9,069 (63.48)	
Yes	17,089 (35.89)	11,872 (35.62)	5,217 (36.52)	
Hypertension				0.243
No	15,443 (32.43)	10,865 (32.60)	4,578 (32.05)	
Yes	32,174 (67.57)	22,466 (67.40)	9,708 (67.95)	
COPD				0.954
No	39,683 (83.34)	27,780 (83.35)	11,903 (83.32)	
Yes	7,934 (16.66)	5,551 (16.65)	2,383 (16.68)	
CG				0.162
No	30,573 (64.21)	21,333 (64.00)	9,240 (64.68)	
Yes	17,044 (35.79)	11,998 (36.00)	5,046 (35.32)	
Hypokalemia				0.713
No	38,564 (80.99)	27,009 (81.03)	11,555 (80.88)	
Yes	9,053 (19.01)	6,322 (18.97)	2,731 (19.12)	
Pulse rate (beats/min)	80.00 (71.00, 90.00)	80.00 (71.00, 90.00)	80.00 (71.00, 91.00)	0.181
SBP (mmHg)	134.00 (122.00, 149.00)	134.00 (122.00, 149.00)	134.00 (122.00, 149.00)	0.957
DBP (mmHg)	80.00 (71.00, 89.00)	80.00 (71.00, 88.00)	80.00 (71.00, 89.00)	0.419
TC (mmol/L)	3.92 (3.25, 4.70)	3.92 (3.24, 4.70)	3.93 (3.25, 4.71)	0.764
TG (mmol/L)	1.2 (0.87, 1.74)	1.20 (0.87, 1.74)	1.20 (0.87, 1.75)	0.562
CREA (*μ*mol/L)	73.00 (59.00, 95.00)	73.00 (59.00, 95.00)	73.00 (59.00, 95.00)	0.509
UA (μmol/L)	339.00 (272.00, 420.00)	338.00 (272.00, 420.00)	339.00 (271.00, 421.00)	0.626
K (mmol/L)	3.83 (3.54, 4.14)	3.83 (3.54, 4.14)	3.83 (3.54, 4.14)	0.924
*P* (mmol/L)	1.10 (0.96, 1.26)	1.10 (0.96, 1.26)	1.10 (0.95, 1.26)	0.044
Cl (mmol/L)	105.00 (102.00, 108.00)	105.00 (102.00, 108.00)	105.00 (102.00, 108.00)	0.932
Na (mmol/L)	141.00 (138.00, 143.00)	141.00 (138.00, 143.00)	141.00 (138.00, 143.00)	0.980
Ca (mmol/L)	2.24 (2.14, 2.33)	2.24 (2.14, 2.33)	2.24 (2.14, 2.32)	0.222
NLR	3.83 (2.50, 6.53)	3.83 (2.50, 6.52)	3.83 (2.49, 6.56)	0.951
PLR	153.54 (112.88, 221.43)	153.60 (113.04, 221.43)	153.31 (112.50, 221.58)	0.944
LMR	3.00 (1.93, 4.31)	3.00 (1.93, 4.32)	2.97 (1.93, 4.29)	0.607
PIV	285.32 (155.61, 592.72)	284.52 (155.6, 591.15)	287.90 (155.72, 597.34)	0.446
FIB-4	2.09 (1.48, 3.08)	2.09 (1.48, 3.07)	2.10 (1.48, 3.10)	0.534
PNI	90.00 (82.65, 99.95)	90.00 (82.65, 99.95)	90.05 (82.66, 99.99)	0.802
FAR	0.26 (0.24, 0.29)	0.26 (0.24, 0.29)	0.26 (0.24, 0.29)	0.079
LDL-C/HDL-C	2.19 (1.59, 2.95)	2.19 (1.59, 2.95)	2.20 (1.60, 2.97)	0.338
NHHR	2.71 (2.03, 3.58)	2.71 (2.03, 3.58)	2.72 (2.03, 3.61)	0.322
CRP/ALB	0.08 (0.01, 0.21)	0.08 (0.01, 0.21)	0.08 (0.01, 0.21)	0.772

HF, heart failure; DM, diabetes mellitus; COPD, chronic obstructive pulmonary disease; CG, chronic gastritis; SBP, systolic blood pressure; DBP, diastolic blood pressure; TC, total cholesterol; TG, triglycerides; CREA, creatinine; UA, uric acid; K, potassium; P, phosphorus; Cl, chloride; Na, sodium; Ca, calcium; NLR, neutrophil-to-lymphocyte ratio; PLR, platelet-to-lymphocyte ratio; LMR, lymphocyte-to-monocyte ratio; PIV, pan-immune-inflammation value; FIB-4, fibrosis-4 index; PNI, prognostic nutritional index; FAR, fibrinogen-to-albumin ratio; LDL-C/HDL-C, low-density lipoprotein cholesterol/high-density lipoprotein cholesterol; NHHR, non-high-density lipoprotein cholesterol to high-density lipoprotein cholesterol ratio; CRP/ALB, c-reactive protein to albumin ratio.

### Predictor screening and multicollinearity assessment

**Figure 1:**
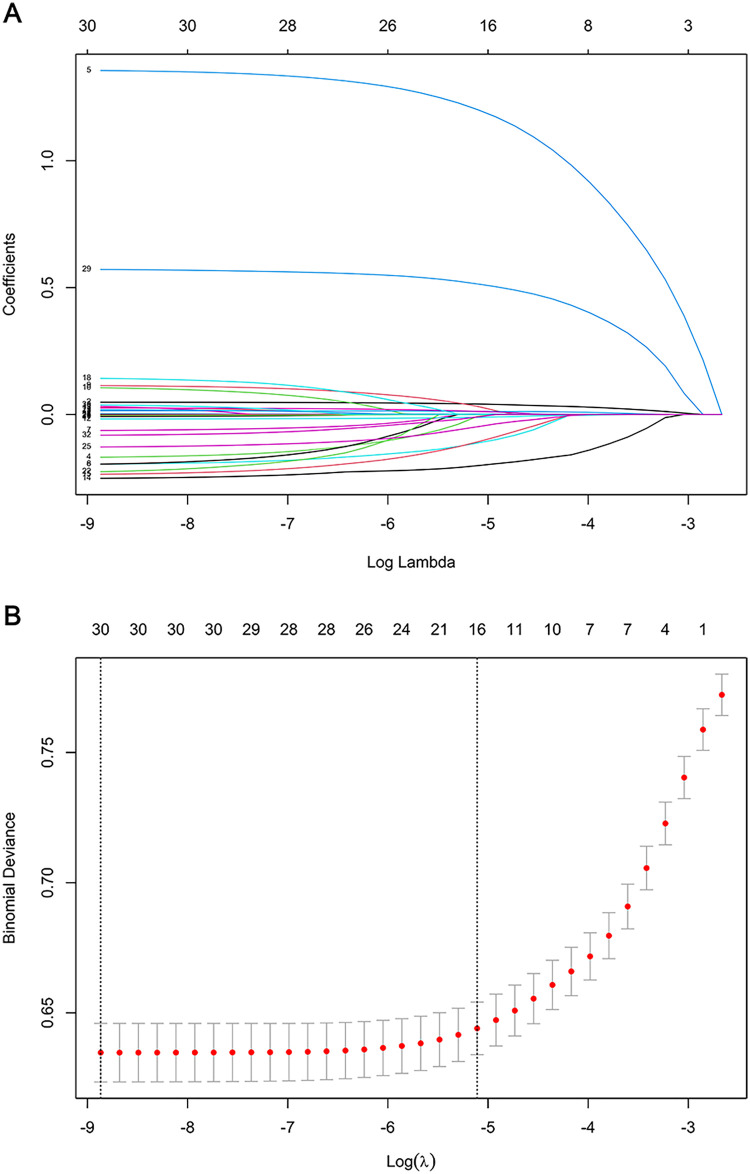
LASSO regression for predictor selection. **(A)** Cross-validation deviance as a function of the log-transformed penalty parameter (*λ*). Vertical dashed lines denote the *λ* value at minimum error (left) and the *λ* at one standard error from the minimum (right, lambda.1se = 0.006045492). **(B)** Coefficient shrinkage paths of the candidate variables. Each curve represents the trajectory of a coefficient as *λ* increases, illustrating the order of variable entry and the effect of regularization strength on model sparsity.

In the training set, all variables except sex (*P* = 0.268), drinking history (*P* = 0.225), surgical history (*P* = 0.205), and PNI (*P* = 0.059) showed significant differences between the AF group and the non-AF group (*P* < 0.05) (Table 2). These statistically significant variables were subsequently entered into LASSO regression analysis. At the optimal penalty coefficient *λ* (lambda.1se = 0.006045492), a total of 16 variables with non-zero coefficients were selected: age, smoking history, HF, DM, CG, pulse rate, SBP, DBP, TC, TG, CREA, UA, PLR, LMR, FAR, and CRP/ALB (Figure 1).

**Table 2:** Between-group comparisons of all variables by AF status in the training set.

Variables	Total (*n* = 33,331)	non-AF group (*n* = 29,001)	AF group (*n* = 4,330)	*P* value
Age (years)	74.00 (66.00, 81.00)	73.00 (66.00, 80.00)	78.00 (73.00, 84.00)	<0.001
Sex				0.268
Male	17,185 (51.56)	14,918 (51.44)	2,267 (52.36)	
female	16,146 (48.44)	14,083 (48.56)	2,063 (47.64)	
Marital status				0.002
married	29,684 (89.06)	25,884 (89.25)	3,800 (87.76)	
unmarried	1,594 (4.78)	1,383 (4.77)	211 (4.87)	
divorced/widowed	2,053 (6.16)	1,734 (5.98)	319 (7.37)	
Smoking history				0.037
No	23,065 (69.20)	20,009 (68.99)	3,056 (70.58)	
Yes	10,266 (30.80)	8,992 (31.01)	1,274 (29.42)	
Drinking history				0.225
No	29,107 (87.33)	25,351 (87.41)	3,756 (86.74)	
Yes	4,224 (12.67)	3,650 (12.59)	574 (13.26)	
Surgical history				0.205
No	24,853 (74.56)	21,590 (74.45)	3,263 (75.36)	
Yes	8,478 (25.44)	7,411 (25.55)	1,067 (24.64)	
HF				<0.001
No	14,809 (44.43)	14,038 (48.41)	771 (17.81)	
Yes	18,522 (55.57)	14,963 (51.59)	3,559 (82.19)	
DM				<0.001
No	21,459 (64.38)	18,453 (63.63)	3,006 (69.42)	
Yes	11,872 (35.62)	10,548 (36.37)	1,324 (30.58)	
Hypertension				<0.001
No	10,865 (32.60)	9,327 (32.16)	1,538 (35.52)	
Yes	22,466 (67.40)	19,674 (67.84)	2,792 (64.48)	
COPD				<0.001
No	27,780 (83.35)	24,342 (83.94)	3,438 (79.40)	
Yes	5,551 (16.65)	4,659 (16.06)	892 (20.60)	
CG				<0.001
No	21,333 (64.00)	18,854 (65.01)	2,479 (57.25)	
Yes	11,998 (36.00)	10,147 (34.99)	1,851 (42.75)	
Hypokalemia				<0.001
No	27,009 (81.03)	23,590 (81.34)	3,419 (78.96)	
Yes	6,322 (18.97)	5,411 (18.66)	911 (21.04)	
Pulse rate (beats/min)	80.00 (71.00, 90.00)	79.00 (71.00, 90.00)	84.00 (73.00, 98.00)	<0.001
SBP (mmHg)	134.00 (122.00, 149.00)	135.00 (122.00, 150.00)	129.00 (116.00, 143.00)	<0.001
DBP (mmHg)	80.00 (71.00, 88.00)	80.00 (72.00, 88.00)	79.00 (70.00, 89.00)	<0.001
TC (mmol/L)	3.92 (3.24, 4.70)	3.99 (3.30, 4.77)	3.54 (2.94, 4.20)	<0.001
TG (mmol/L)	1.20 (0.87, 1.74)	1.24 (0.89, 1.80)	1 (0.76, 1.38)	<0.001
CREA (μmol/L)	73.00 (59.00, 95.00)	72.00 (59.00, 92.00)	81.00 (64.00, 110.00)	<0.001
UA (μmol/L)	338.00 (272.00, 420.00)	334.00 (269.00, 412.00)	374 (295, 475)	<0.001
K (mmol/L)	3.83 (3.54, 4.14)	3.82 (3.53, 4.12)	3.90 (3.57, 4.26)	<0.001
*P* (mmol/L)	1.10 (0.96, 1.26)	1.10 (0.96, 1.26)	1.11 (0.96, 1.29)	0.017
Cl (mmol/L)	105.00 (102.00, 108.00)	105.00 (103.00, 108.00)	105.00 (102.00, 108.00)	0.003
Na (mmol/L)	141.00 (138.00, 143.00)	141.00 (138.00, 143.00)	140.00 (138.00, 142.48)	<0.001
Ca (mmol/L)	2.24 (2.14, 2.33)	2.24 (2.15, 2.33)	2.21 (2.12, 2.30)	<0.001
NLR	3.83 (2.50, 6.52)	3.71 (2.44, 6.30)	4.69 (3.02, 8.10)	<0.001
PLR	153.60 (113.04, 221.43)	152.75 (112.99, 218.40)	160.18 (113.65, 237.78)	<0.001
LMR	3.00 (1.93, 4.32)	3.10 (2.00, 4.44)	2.36 (1.54, 3.44)	<0.001
PIV	284.52 (155.6, 591.15)	277.54 (152.65, 576.55)	338.02 (179.19, 698.36)	<0.001
FIB-4	2.09 (1.48, 3.07)	2.02 (1.44, 2.91)	2.79 (1.93, 4.05)	<0.001
PNI	90.00 (82.65, 99.95)	90.05 (82.80, 99.85)	89.70 (81.60, 100.99)	0.059
FAR	0.26 (0.24, 0.29)	0.26 (0.24, 0.29)	0.28 (0.25, 0.32)	<0.001
LDL-C/HDL-C	2.19 (1.59, 2.95)	2.22 (1.61, 2.97)	2.02 (1.43, 2.82)	<0.001
NHHR	2.71 (2.03, 3.58)	2.74 (2.06, 3.61)	2.51 (1.84, 3.31)	<0.001
CRP/ALB	0.08 (0.01, 0.21)	0.07 (0.01, 0.19)	0.11 (0.02, 0.30)	<0.001

HF, heart failure; DM, diabetes mellitus; COPD, chronic obstructive pulmonary disease; CG, chronic gastritis; SBP, systolic blood pressure; DBP, diastolic blood pressure; TC, total cholesterol; TG, triglycerides; CREA, creatinine; UA, uric acid; K, potassium; P, phosphorus; Cl, chloride; Na, sodium; Ca, calcium; NLR, neutrophil-to-lymphocyte ratio; PLR, platelet-to-lymphocyte ratio; LMR, lymphocyte-to-monocyte ratio; PIV, pan-immune-inflammation value; FIB-4, fibrosis-4 index; PNI, prognostic nutritional index; FAR, fibrinogen-to-albumin ratio; LDL-C/HDL-C, low-density lipoprotein cholesterol/high-density lipoprotein cholesterol; NHHR, non-high-density lipoprotein cholesterol to high-density lipoprotein cholesterol ratio; CRP/ALB, c-reactive protein to albumin ratio.

**Figure 2:**
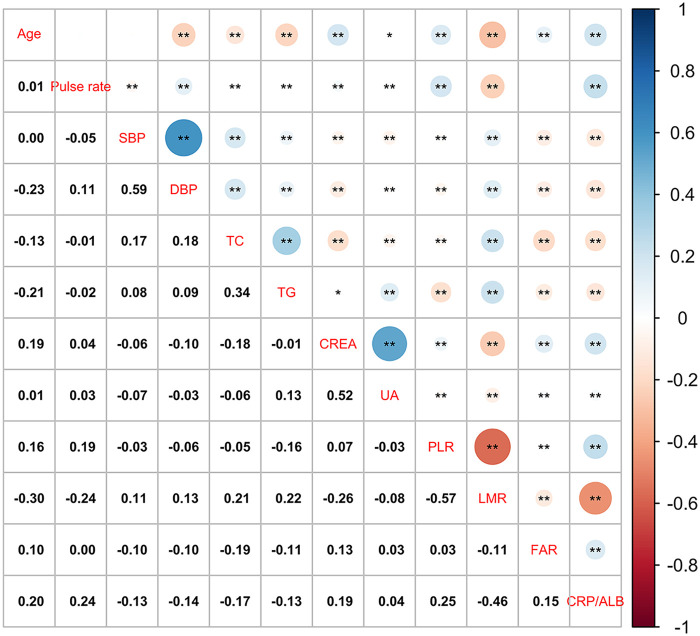
Spearman correlation heatmap of LASSO-selected continuous predictors.

To comprehensively evaluate multicollinearity among these 16 LASSO-selected predictors, correlation analysis and collinearity diagnostics were performed. As shown in Figure 2, the absolute values of all Spearman correlation coefficients between continuous variables did not exceed 0.60. The strongest correlation was observed between SBP and DBP (*r* = 0.59), followed by PLR and LMR (*r* = –0.57). In addition, CREA and UA also showed a moderately strong positive correlation (*r* = 0.52). Supplementary Table 6 further presents the VIF and tolerance for each variable. The VIF values ranged from 1.017 to 1.976, well below the conventional threshold of 5.

### Nonlinear association patterns of continuous predictors

**Figure 3:**
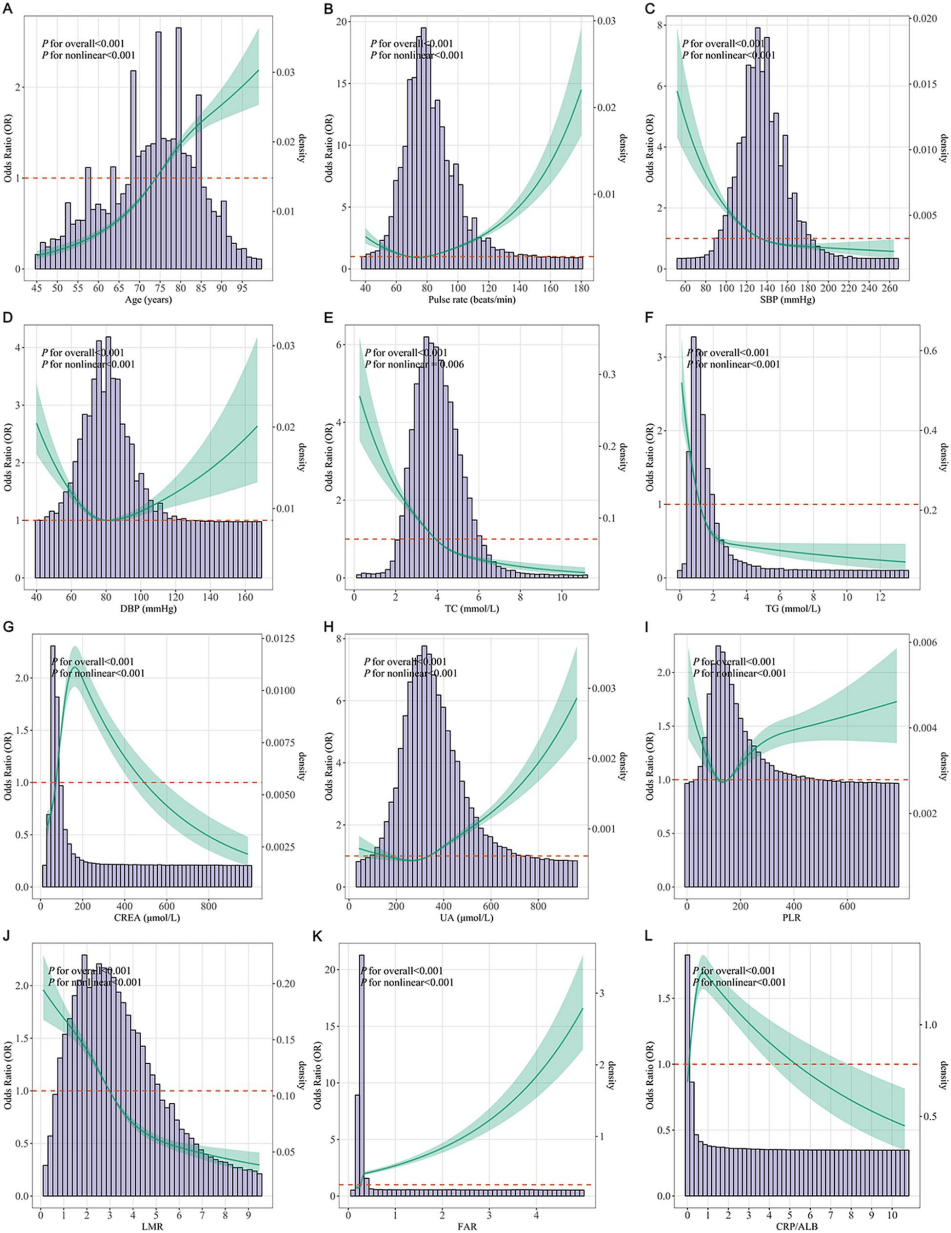
Dose-response relationships between LASSO-selected continuous variables and AF risk based on restricted cubic spline (RCS) regression. **(A)** Age; **(B)** pulse rate; **(C)** SBP; **(D)** DBP; **(E)** TC; **(F)** TG; **(G)** CREA; **(H)** UA; **(I)** PLR; **(J)** LMR; **(K)** FAR; **(L)** CRP/ALB.

To investigate the dose-response relationships between the 12 continuous variables selected by LASSO and the risk of AF, RCS regression was performed (Figure 3). Distinct patterns of association emerged across the variables. Specifically, both age (*P* for nonlinear < 0.001) and FAR (*P* for nonlinear < 0.001) exhibited nonlinear positive dose-response relationships, whereas TC (*P* for nonlinear = 0.006) and LMR (*P* for nonlinear < 0.001) showed nonlinear negative dose-response relationships. Pulse rate, DBP, and PLR each demonstrated a U-shaped association (all *P* for nonlinear < 0.001). SBP, TG, and UA each exhibited a J-shaped association (all *P* for nonlinear < 0.001). In contrast, CREA and CRP/ALB displayed an inverted U-shaped association (both *P* for nonlinear < 0.001).

### Model development and predictive performance assessment

**Figure 4:**
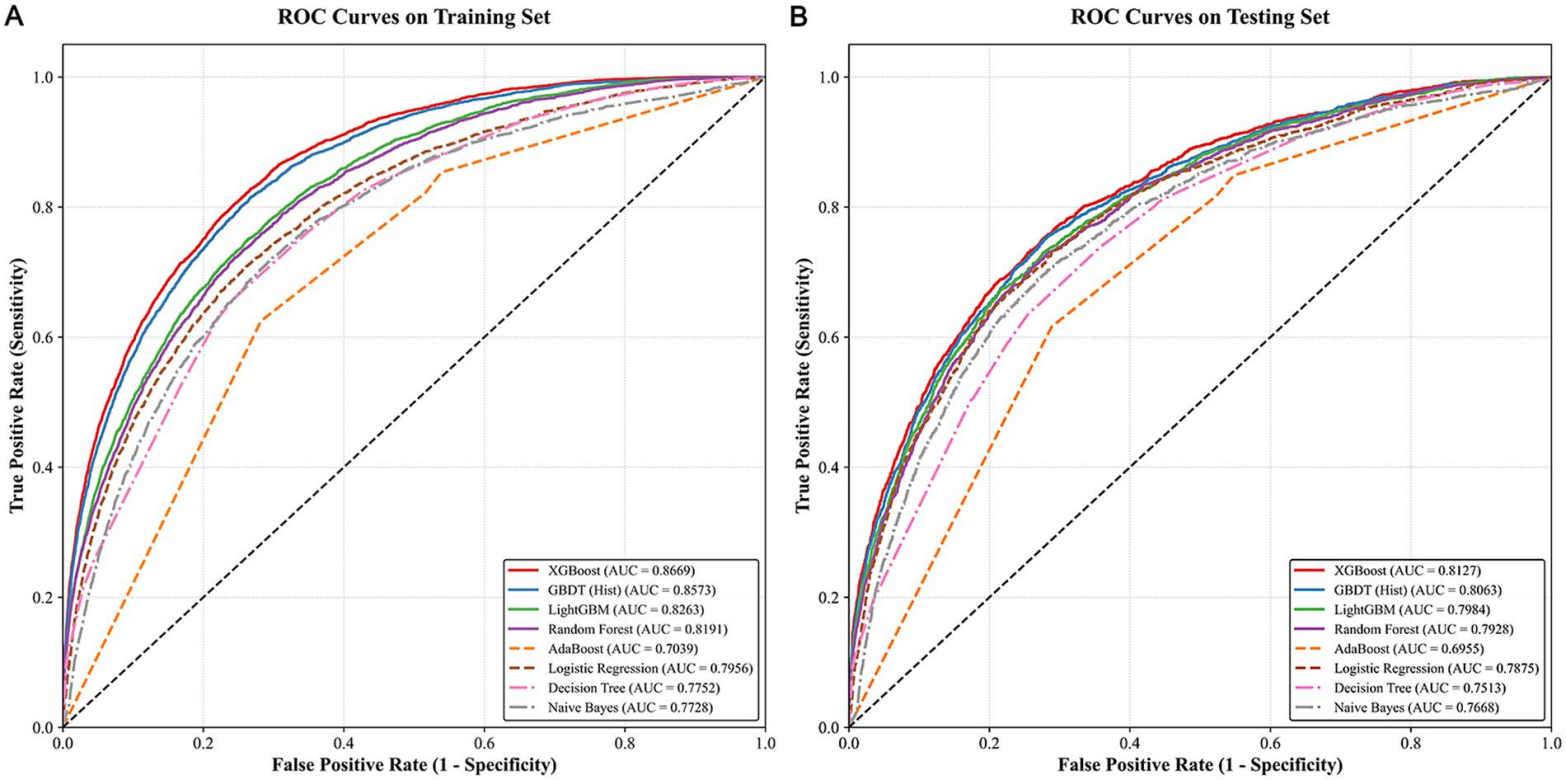
Receiver operating characteristic (ROC) curves and area under the curve (AUC) of eight ML models. **(A)** Training set; **(B)** validation set.

**Figure 5:**
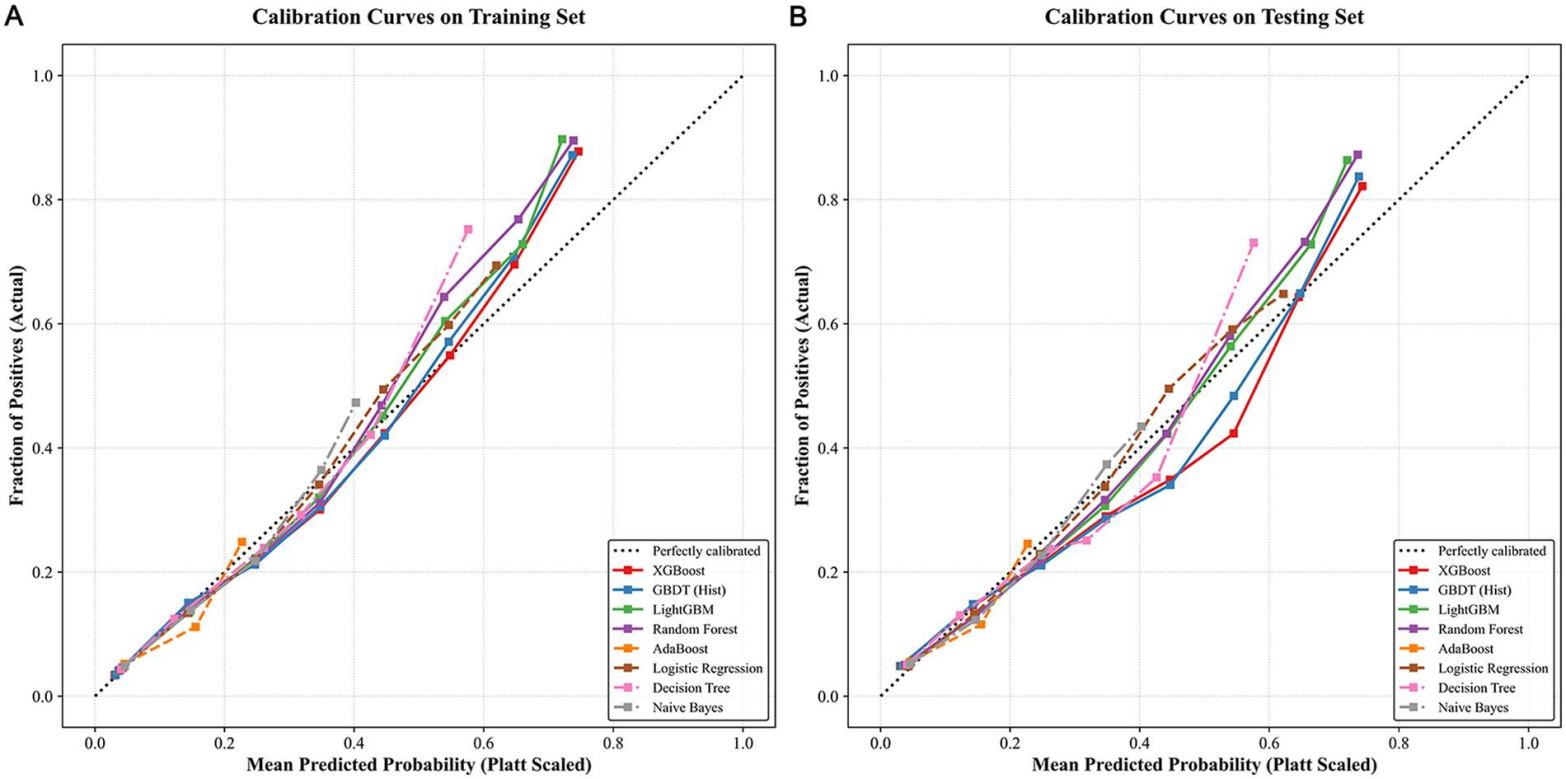
Calibration curves for predicted versus observed AF risk across eight ML models. **(A)** Training set; **(B)** validation set.

**Figure 6:**
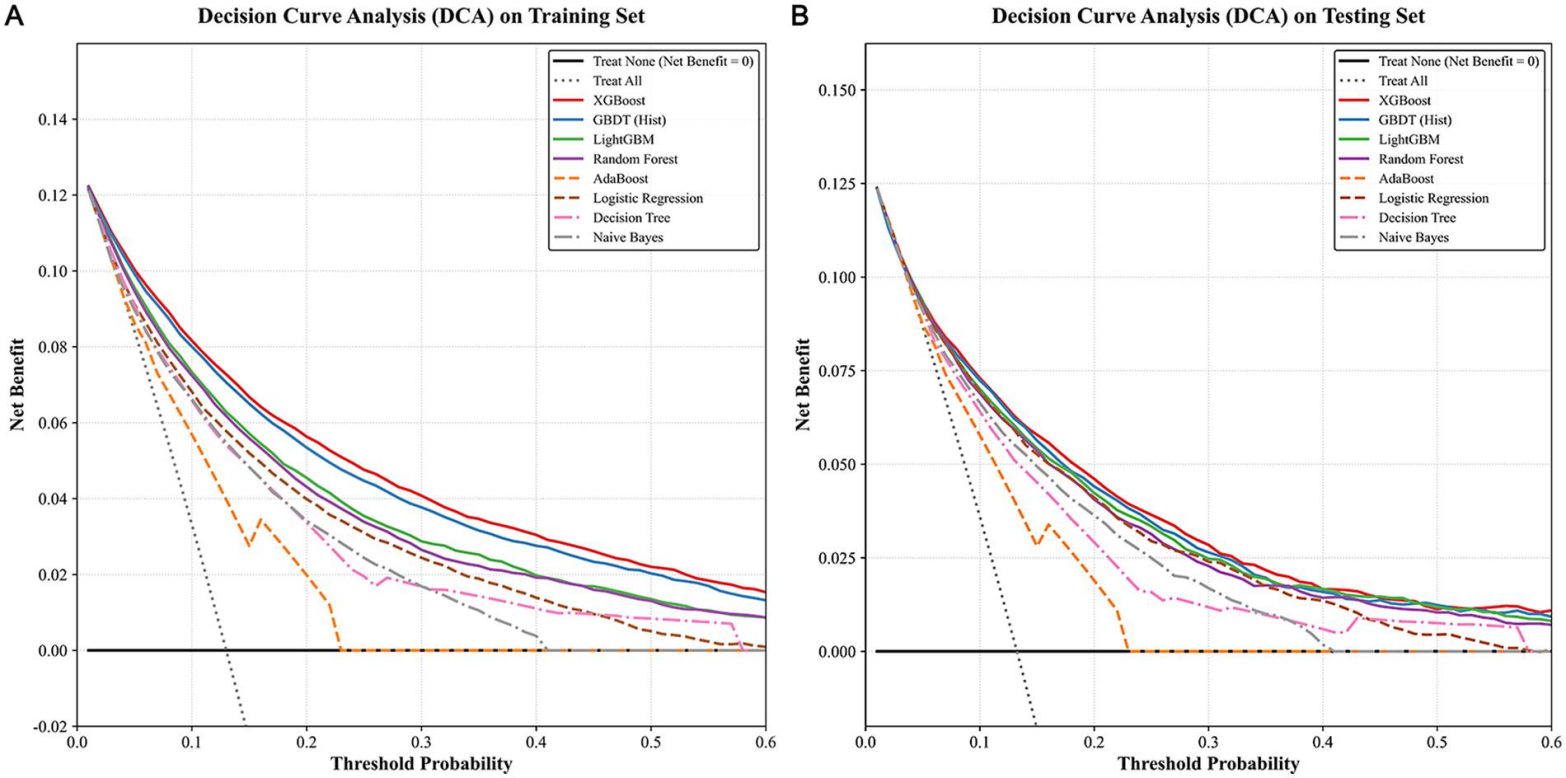
Decision curve analysis (DCA) comparing the net benefit of eight ML models across a range of threshold probabilities. **(A)** Training set; **(B)** validation set.

Based on 16 predictors identified by LASSO regression, eight machine learning models were developed to assess the risk of coexisting AF in middle-aged and older patients with CHD. Predictive performance was evaluated separately in the training and validation cohorts. Figure 4 presents the AUC values of the eight models across both datasets. Meanwhile, Tables 3, 4 summarize four performance metrics—sensitivity, specificity, F1 score, and Youden index—for the training and validation sets, respectively. Among the eight models, XGBoost achieved superior predictive performance in both cohorts. Specifically, the XGBoost model yielded an AUC of 0.867 (95% CI: 0.862–0.872) in the training set and 0.813 (95% CI: 0.802–0.823) in the validation set, with corresponding sensitivity values of 0.789 and 0.714, specificity of 0.770 and 0.755, Youden index of 0.559 and 0.470, and F1 scores of 0.474 and 0.430. Furthermore, calibration curves (Figure 5) and DCA (Figure 6) provided empirical support for selecting the optimal predictive tool in clinical practice.

**Table 3:** Predictive performance of eight ML models for AF risk in the training set.

Model	AUC (95%CI)	Sensitivity	Specificity	Youden index	F1 Score
XGBoost	0.867 (0.862–0.872)	0.789	0.770	0.559	0.474
GBDT	0.857 (0.852–0.863)	0.783	0.761	0.544	0.463
LightGBM	0.826 (0.820–0.833)	0.762	0.721	0.483	0.420
RF	0.819 (0.813–0.826)	0.752	0.725	0.476	0.418
AdaBoost	0.704 (0.697–0.711)	0.822	0.484	0.306	0.311
LR	0.796 (0.789–0.803)	0.733	0.712	0.444	0.400
DT	0.775 (0.768–0.783)	0.658	0.758	0.416	0.401
NB	0.773 (0.765–0.781)	0.742	0.678	0.420	0.381

AUC, area under the ROC curve; CI, confidence interval; XGBoost, extreme Gradient Boosting; GBDT, gradient boosting decision tree; LightGBM, light gradient boosting machine; RF, random forest; AdaBoost, adaptive boosting; LR, logistic regression; DT, decision tree; NB, naive Bayes.

**Table 4:** Predictive performance of eight ML models for AF risk in the validation set.

Model	AUC (95%CI)	Sensitivity	Specificity	Youden index	F1 Score
XGBoost	0.813 (0.802–0.823)	0.714	0.755	0.470	0.430
GBDT	0.806 (0.796–0.817)	0.721	0.745	0.466	0.425
LightGBM	0.798 (0.787–0.809)	0.739	0.709	0.448	0.405
RF	0.793 (0.781–0.803)	0.731	0.714	0.445	0.405
AdaBoost	0.696 (0.684–0.707)	0.817	0.477	0.294	0.312
LR	0.788 (0.776–0.798)	0.736	0.704	0.440	0.401
DT	0.751 (0.739–0.762)	0.637	0.744	0.381	0.384
NB	0.767 (0.755–0.778)	0.737	0.672	0.409	0.379

AUC, area under the ROC curve; CI, confidence interval; XGBoost, extreme Gradient Boosting; GBDT, gradient boosting decision tree; LightGBM, light gradient boosting machine; RF, random forest; AdaBoost, adaptive boosting; LR, logistic regression; DT, decision tree; NB, naive Bayes.

In the stratified 5-fold cross-validation on the training set, XGBoost achieved a mean AUC of 0.812 (SD: 0.005), demonstrating stable performance across folds (fold-wise AUCs: 0.815, 0.811, 0.818, 0.811, and 0.804). GBDT achieved a mean AUC of 0.809 (SD: 0.005), LightGBM achieved 0.800 (SD: 0.006), RF achieved 0.795 (SD: 0.007), LR achieved 0.795 (SD: 0.005), NB achieved 0.773 (SD: 0.010), DT achieved 0.757 (SD: 0.005), and AdaBoost achieved 0.699 (SD: 0.008). Paired comparisons using the Delong test revealed that XGBoost significantly outperformed all other seven models on the validation set (all FDR-adjusted *P* < 0.001). In the context of class imbalance, the XGBoost model achieved an AF-class F1-score of 0.432 and a non-AF-class F1-score of 0.840. While the AF-class recall (sensitivity) was 0.718, the precision was notably lower at 0.309. The model maintained high precision (0.946) and recall (0.756) for the non-AF class. Detailed per-class performance metrics for all eight models on the validation set are presented in Supplementary Table 7, and fold-wise cross-validation results for all eight models are provided in Supplementary Table 8.

Supplementary Figure 2 presents the confusion matrices and Precision-Recall (PR) curves for the XGBoost model on the training and validation sets. On the validation set, the model correctly classified 1,357 AF cases (true positives) and 9,363 non-AF cases (true negatives), with 3,032 false negatives and 534 false positives. The PR curves yielded an average precision (AP) of 0.554 on the training set and 0.474 on the validation set, reflecting the model's ability to maintain precision in the context of class imbalance.

### Feature importance quantification via SHAP analysis

**Figure 7:**
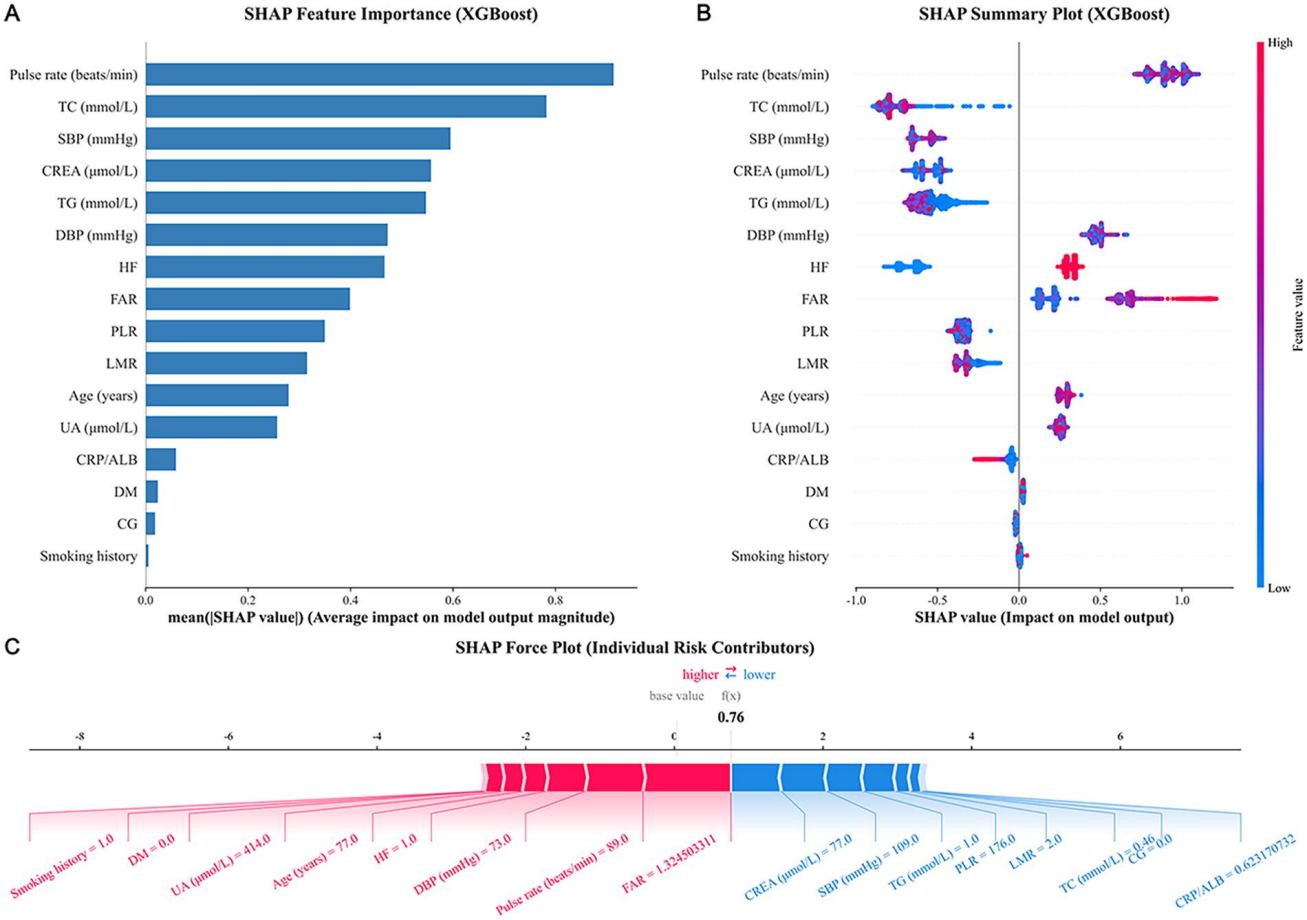
SHAP (shapley additive explanations) analysis for interpreting the XGBoost model. **(A)** SHAP-based feature importance bar chart. **(B)** SHAP summary dot plot: red and blue colors indicate high and low feature values, respectively; SHAP values to the left of the vertical axis decrease predicted AF risk, while those to the right increase it. **(C)** Force plot for an individual patient, demonstrating the contribution of each feature to the patient-specific risk prediction.

Figure 7A displays the variable importance ranking within the XGBoost model based on SHAP values, with the top five predictors being pulse rate, TC, SBP, CREA, and TG. Quantitatively, pulse rate had the highest mean absolute SHAP value of 1.036, followed by TC (0.784), SBP (0.598), CREA (0.506), and TG (0.503), indicating their dominant contributions to the model's predictions. The mean absolute SHAP values for all 16 LASSO-selected predictors are provided in Supplementary Table 9. Figure 7B presents the SHAP summary plot, in which red and blue denote higher and lower feature values, respectively. A SHAP value below zero indicates a negative contribution to the prediction, whereas a value above zero reflects a positive effect. The dispersion of each feature along the *Y*-axis is directly associated with its relative importance. Figure 7C further illustrates the SHAP force plot for representative patients, detailing the direction and magnitude of each feature's contribution to individual predictions.

### Local interpretability analysis using LIME

**Figure 8:**
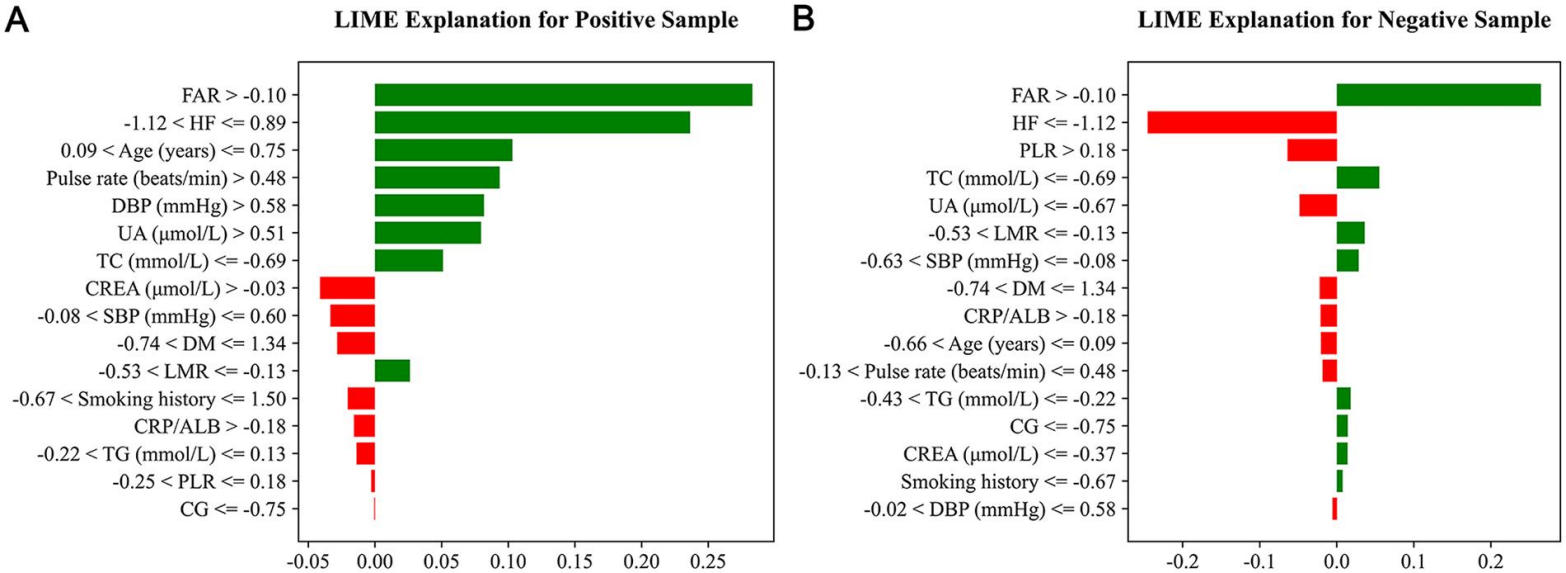
LIME (local interpretable model-agnostic explanations) analysis for individual patient predictions from the XGBoost model. **(A)** Feature contributions for a positive case. **(B)** Feature contributions for a negative case. Feature values are standardized; green/red bars indicate increased/decreased AF risk relative to the local baseline.

To complement the global SHAP analysis, we employed LIME to examine the feature contributions for individual patient predictions. Figure 8 illustrates two representative cases from the validation set. Figure 8A illustrates a positive case where the top three risk-enhancing features were FAR > −0.10 (contribution: ∼0.28), HF within the range of −1.12 to 0.89 (contribution: ∼0.24), and age between 0.09 and 0.75 years (contribution: ∼0.10), collectively driving the prediction toward AF; conversely, protective features including CREA >−0.03, SBP between −0.08 and 0.60 mmHg, and DM between −0.74 and 1.34 exerted minor negative contributions. Figure 8B presents a negative case characterized by predominantly protective indicators, with the strongest protective features being DBP ≤0.58 mmHg, smoking history ≤−0.67, and CREA ≤−0.37 μmol/L, alongside HF ≤ −1.12 and FAR ≤−0.10 as the most influential risk-reducing variables, resulting in an overall low AF probability.

### Ablation analysis

The ablation results are summarized in Supplementary Table 10. In the feature ablation experiment, sequential removal of the top-five SHAP-ranked predictors resulted in progressive performance degradation: removal of pulse rate caused the largest drop in AUC (from 0.813 to 0.796, Δ = −0.017), followed by TC (Δ = −0.012), SBP (Δ = −0.010), CREA (Δ = −0.008), and TG (Δ = −0.004), with cumulative removal of all five features reducing the AUC to 0.784 (Δ = −0.029). In the preprocessing ablation experiment, RF imputation yielded the highest AUC (0.813), outperforming mean/median imputation (0.792, Δ = −0.021) and complete-case analysis (0.801, Δ = −0.012). In the optimizer ablation experiment, the grid search-optimized model achieved an AUC of 0.813, substantially outperforming the default-parameter model (0.787, Δ = −0.026).

## Discussion

In this retrospective study of over 47,000 middle-aged and older CHD patients, we systematically developed and compared eight machine learning models for AF risk prediction. Among them, XGBoost consistently outperformed the alternatives across both training and validation cohorts, as reflected by its superior AUC, sensitivity, and Youden index. This advantage likely stems from XGBoost's inherent capacity to handle nonlinear relationships, capture higher-order interactions among predictors, and regularize model complexity, thereby reducing overfitting—a common pitfall in clinical prediction modeling. Beyond predictive accuracy, we incorporated SHAP analysis to enhance model transparency, allowing clinicians to visualize both the direction and magnitude of each feature's contribution to individual risk estimates. Taken together, these findings suggest that a machine learning framework, particularly XGBoost combined with SHAP interpretation, can serve as a practical and evidence-based tool for AF risk assessment in the CHD population.

Among the top five predictors identified by SHAP analysis—pulse rate, TC, SBP, CREA, and TG—each has been independently linked to AF risk in previous studies, yet their combined contribution within a nonlinear machine learning framework has rarely been quantified (24–27). Elevated pulse rate, often reflecting autonomic dysregulation or subclinical cardiac stress, may promote atrial electrical remodeling and ectopic activity, thereby increasing AF susceptibility (28). Likewise, TC and TG, as conventional lipid markers, have shown inconsistent associations with AF in traditional regression models; however, our findings suggest that their predictive value emerges when nonlinear interactions with other metabolic factors are jointly modeled. SBP, a well-established risk factor for cardiovascular events, may contribute to AF through left atrial pressure overload and subsequent structural remodeling (29). Elevated CREA, indicative of reduced renal function, is known to disturb fluid and electrolyte homeostasis, potentially amplifying atrial vulnerability (30). The prominence of these five variables underscores the multifactorial nature of AF pathogenesis in CHD patients, spanning hemodynamic, metabolic, and renal domains.

Beyond the top five contributors, several other LASSO-selected predictors—particularly PLR, LMR, FAR, and CRP/ALB—merit discussion due to their reflection of systemic inflammation and nutritional status. PLR and LMR, derived from routine blood counts, serve as surrogates for the balance between pro-inflammatory and anti-inflammatory pathways. In our model, lower LMR and higher PLR were associated with increased AF risk, consistent with the hypothesis that a heightened inflammatory state facilitates atrial fibrosis and electrical instability (31, 32). FAR and CRP/ALB, integrating coagulation, inflammatory, and nutritional signals, have recently emerged as prognostic markers in various cardiovascular conditions (33, 34). Their inclusion in the LASSO-derived feature set further reinforces the notion that AF development in CHD patients is not solely driven by traditional hemodynamic factors but is also modulated by low-grade systemic inflammation and protein metabolism disturbances. Notably, the fact that these composite inflammatory markers survived dimensionality reduction—while some conventional variables did not—highlights their potential added value in risk stratification.

The restricted cubic spline analyses yielded several nonlinear dose-response patterns that carry both pathophysiological and clinical implications. Age and FAR exhibited nonlinear positive associations with AF risk, suggesting that incremental increases in these variables—particularly beyond certain thresholds—may disproportionately elevate risk, rather than following a simple linear gradient. Conversely, TC and LMR displayed nonlinear negative associations, indicating that lower levels of these markers are linked to higher AF probability, a finding that may appear counterintuitive but could reflect reverse causality or malnutrition in high-risk subgroups (35). More striking were the U-shaped (pulse rate, DBP, PLR) and J-shaped (SBP, TG, UA) associations, which imply that both low and high extremes of these physiological parameters confer elevated AF risk, whereas moderate levels are relatively protective (36, 37). This nonlinear complexity may explain why conventional logistic regression models, which assume linearity, often yield inconsistent or null findings for these variables. From a clinical perspective, these patterns argue against a “one-size-fits-all” risk target and instead support individualized optimization of each parameter within a favorable range.

The superior performance of XGBoost relative to logistic regression and other conventional classifiers merits methodological reflection. Ensemble gradient boosting algorithms excel in handling high-dimensional, heterogeneous data with intricate interaction structures—precisely the architecture of EMR-derived clinical variables (38, 39). By sequentially correcting residuals across decision trees, XGBoost effectively models the hierarchical, non-additive effects that likely underlie AF pathogenesis in CHD patients. However, raw predictive accuracy alone is insufficient for clinical adoption; the SHAP framework addresses this by decomposing individual predictions into additive, directionally signed feature contributions. For instance, a clinician encountering a patient with tachycardia, elevated FAR, and marginal renal function can visualize how each factor pushes the predicted probability upward, facilitating shared decision-making around anticoagulation initiation or upstream risk-factor modification. To complement SHAP's global and locally consistent explanations, we additionally employed LIME to provide intuitive decision-boundary interpretations at the individual case level; the two frameworks showed consistent feature contributions, further reinforcing the clinical interpretability of the model. Calibration curves and decision curve analysis further buttress clinical utility: the model maintains probability fidelity across the risk spectrum and delivers positive net benefit across a clinically plausible range of threshold probabilities. Looking ahead, integration of this model into computerized physician order entry systems could generate real-time AF risk alerts at the point of care, potentially triggering protocolized screening with ambulatory electrocardiography or biomarker surveillance in flagged high-risk individuals.

The class imbalance inherent in AF prediction (AF vs. non-AF ratio ≈ 1:7) warrants specific consideration. While we elected not to apply oversampling or synthetic augmentation to preserve the natural prevalence distribution that reflects real-world clinical settings, we employed two complementary strategies to address this issue: (1) stratified 5-fold cross-validation to maintain consistent class proportions across folds, ensuring that model evaluation was not affected by arbitrary variations in class distribution; and (2) comprehensive per-class performance reporting to transparently evaluate model performance on both AF and non-AF cases. The moderate AF-class precision observed in our study (0.309) reflects the inherent trade-off when predicting a minority class without explicit imbalance correction. This pattern of performance is clinically acceptable in the context of AF screening, where positive predictions trigger relatively low-risk confirmatory testing (e.g., ECG monitoring), and the clinical priority is to achieve high sensitivity while maintaining acceptable specificity. A positive model output should therefore prompt confirmatory diagnostic testing rather than immediate therapeutic intervention (40). We acknowledge that alternative strategies—including class-weighted loss functions, SMOTE, or cost-sensitive learning—may further improve minority-class recall or precision; however, such approaches risk introducing artificial data distributions that may not generalize to real-world settings. Future work should systematically compare multiple imbalance strategies to identify optimal handling methods for AF risk prediction in CHD populations.

The ablation analyses further substantiate the model's component contributions. The feature ablation experiments confirmed that the top-five SHAP-ranked predictors collectively account for a substantial proportion of predictive performance (cumulative AUC reduction of 0.055), with pulse rate and TC exerting the strongest individual contributions. This aligns with the clinical literature identifying hemodynamic and lipid parameters as major AF risk determinants in CHD patients (3, 28). The preprocessing ablation revealed that random forest imputation, though computationally more expensive, provides a measurable improvement in predictive accuracy, supporting its use in EMR-based modeling where missing data are inevitable (41). The optimizer ablation highlighted the importance of systematic hyperparameter tuning, as default parameters yielded considerably lower performance. Collectively, these findings demonstrate that each component of our modeling pipeline contributes meaningfully to the final predictive performance.

While deep learning architectures—particularly attention-based and transformer models—have achieved remarkable success in ECG signal analysis for AF detection, their application to structured tabular EMR data warrants careful consideration (42, 43). A recent systematic review and meta-analysis comparing machine learning and deep learning prognostic models in AF found that deep learning achieved higher pooled AUCs than machine learning in AF recurrence (0.82 vs. 0.78) and stroke (0.80 vs. 0.75) (44). However, when trained on the same datasets, both approaches consistently outperformed traditional clinical risk scores, and the performance advantage of deep learning was not universally consistent across all tasks (44). Furthermore, a comparative study on imbalanced clinical tabular data demonstrated that tree-based ensemble methods, particularly XGBoost, consistently achieved the most stable performance across imbalance levels and scaled efficiently with sample size, whereas deep tabular models (e.g., TabNet) degraded more sharply under imbalance and incurred substantially higher computational costs (45). Given that our data are structured tabular variables rather than raw ECG signals, and considering the practical constraints of clinical deployment—including inference speed, hardware requirements, and interpretability—the XGBoost model represents a pragmatic balance between predictive performance and real-world feasibility. Nevertheless, we acknowledge that systematic benchmarking against attention-based tabular architectures (e.g., TabNet, FT-Transformer) represents an important direction for future work, particularly as larger multi-center EMR datasets become available for training deep models (46).

Translating our model into clinical practice requires systematic consideration of implementation, regulatory, ethical, and technical dimensions (47). For deployment, the model can be integrated into EMR systems via APIs as a clinical decision support tool, with clinicians retaining final judgment. Regulatory classification as a Software as a Medical Device (SaMD) would require prospective validation, transparent documentation, and post-market monitoring; our SHAP/LIME framework directly addresses transparency mandates (e.g., EU AI Act) (48, 49). Ethical safeguards should include algorithmic fairness audits across subgroups, compliance with privacy regulations, and a shared accountability model for liability. To address device heterogeneity across institutions, we propose automated data quality checks, site-specific calibration, and continuous performance monitoring. The three-phase validation roadmap includes: temporal validation on a recent cohort from the same institution (Phase I), geographic validation on datasets from three additional hospitals (Phase II, currently in data collection as described in Limitations), and prospective deployment at one site assessing discrimination, calibration, and clinical utility (Phase III).

Several limitations should be acknowledged. First, the data were derived from a single institution, which may limit the generalizability of our findings to broader populations or healthcare systems with different demographic and clinical profiles. Although all eligible patients were consecutively enrolled and diagnostic criteria were standardized, the institutional-specific patient mix and treatment patterns may introduce bias. We have therefore planned a multicenter external validation study involving three additional hospitals in Chongqing and Sichuan provinces, which is currently in the data collection phase. This will be the focus of our subsequent work. Second, despite the use of random forest imputation, the retrospective nature of electronic medical record data inevitably introduces potential selection bias and information bias. We acknowledge that missing data may not be completely at random, and that imputation could introduce bias if missingness is associated with disease severity or healthcare utilization patterns. Future work should explore multiple imputation methods and sensitivity analyses to assess the robustness of our findings to different imputation strategies. Third, although we incorporated a wide range of clinical and laboratory variables, unmeasured confounders—such as medication adherence, lifestyle modifications, or genetic factors—could not be accounted for. Lastly, the SHAP analysis, while informative, provides global and local interpretability at a static prediction level rather than offering dynamic, time-updated risk estimation.

Several directions warrant further exploration. Multi-modal integration could incorporate 12-lead ECG signals, echocardiographic parameters, or cardiac imaging features with structured clinical data to enhance predictive performance. Longitudinal studies with repeated measurements would enable dynamic risk prediction, capturing temporal changes that better reflect AF pathogenesis than static assessments. Federated learning offers a paradigm for privacy-preserving multi-center collaboration, improving generalizability across diverse populations. Prospective clinical trials—particularly cluster-randomized or stepped-wedge designs—are essential to establish whether model-guided screening improves AF detection and clinical outcomes. Finally, a user-friendly interface, such as a web-based calculator or EMR-integrated dashboard, will facilitate real-time bedside risk assessment. These directions collectively aim to transform our model into a mature, clinically deployable tool.

## Conclusions

In summary, this study demonstrates that an XGBoost-based model built on 16 readily available clinical predictors achieves robust predictive performance for AF among middle-aged and older CHD patients. The integration of SHAP analysis bridges the gap between model accuracy and clinical interpretability, addressing a key barrier to the adoption of machine learning in routine practice. Future efforts should focus on external validation, prospective deployment, and the development of a user-friendly interface—such as a web-based or bedside risk calculator—to facilitate real-time, individualized risk assessment and support timely preventive interventions.

## Data Availability

The raw data supporting the conclusions of this article will be made available by the authors, without undue reservation.
