# Supplementary material for: Development and validation of an interpretable machine learning model for predicting atrial fibrillation risk in middle-aged and older patients with coronary heart disease
Source: Front Cardiovasc Med. 2026 Jul 8;13:1886992. doi: 10.3389/fcvm.2026.1886992 (PMC13388312; doi:10.3389/fcvm.2026.1886992)
Supplement: Supplementary file 1 [file Supplementaryfile1.docx]

**Table S1.** Missing proportions of all variables in the training set

| **Variables** | **Missing count (n)** | **Missing proportion (%)** |
| --- | --- | --- |
| Age (years) | 0 | 0 |
| Sex | 0 | 0 |
| Marital status | 0 | 0 |
| Smoking history | 0 | 0 |
| Drinking history | 0 | 0 |
| Surgical history | 0 | 0 |
| HF | 0 | 0 |
| DM | 0 | 0 |
| Hypertension | 0 | 0 |
| COPD | 0 | 0 |
| CG | 0 | 0 |
| Hypokalemia | 0 | 0 |
| Pulse rate (beats/min) | 275 | 0.83 |
| SBP (mmHg) | 6111 | 18.33 |
| DBP (mmHg) | 6134 | 18.40 |
| TC (mmol/L) | 6399 | 19.20 |
| TG (mmol/L) | 6471 | 19.41 |
| CREA (μmol/L) | 2095 | 6.29 |
| UA (μmol/L) | 2096 | 6.29 |
| K (mmol/L) | 1538 | 4.61 |
| P (mmol/L) | 6934 | 20.80 |
| Cl (mmol/L) | 1537 | 4.61 |
| Na (mmol/L) | 1537 | 4.61 |
| Ca (mmol/L) | 5991 | 17.97 |
| NLR | 0 | 0 |
| PLR | 0 | 0 |
| LMR | 0 | 0 |
| PIV | 0 | 0 |
| FIB-4 | 0 | 0 |
| PNI | 0 | 0 |
| FAR | 0 | 0 |
| LDL-C/HDL-C | 0 | 0 |
| NHHR | 0 | 0 |
| CRP/ALB | 0 | 0 |

Abbreviations: **HF**, heart failure; **DM**, diabetes mellitus; **COPD**, chronic obstructive pulmonary disease; **CG**, chronic gastritis; **SBP**, systolic blood pressure; **DBP**, diastolic blood pressure; **TC**, total cholesterol; **TG**, triglycerides; **CREA**, creatinine; **UA**, uric acid; **K**, potassium; **P**, phosphorus; **Cl**, chloride; **Na**, sodium; **Ca**, calcium; **NLR**, neutrophil-to-lymphocyte ratio; **PLR**, platelet-to-lymphocyte ratio; **LMR**, lymphocyte-to-monocyte ratio; **PIV**, pan-immune-inflammation value; **FIB-4**, fibrosis-4 index; **PNI**, prognostic nutritional index; **FAR**, fibrinogen-to-albumin ratio; **LDL-C/HDL-C**, low-density lipoprotein cholesterol/high-density lipoprotein cholesterol; **NHHR**, non-high-density lipoprotein cholesterol to high-density lipoprotein cholesterol ratio; **CRP/ALB**, c-reactive protein to albumin ratio.

**Table S2.** Missing proportions of all variables in the validation set

| **Variables** | **Missing count (n)** | **Missing proportion (%)** |
| --- | --- | --- |
| Age (years) | 0 | 0 |
| Sex | 0 | 0 |
| Marital status | 0 | 0 |
| Smoking history | 0 | 0 |
| Drinking history | 0 | 0 |
| Surgical history | 0 | 0 |
| HF | 0 | 0 |
| DM | 0 | 0 |
| Hypertension | 0 | 0 |
| COPD | 0 | 0 |
| CG | 0 | 0 |
| Hypokalemia | 0 | 0 |
| Pulse rate (beats/min) | 130 | 0.91 |
| SBP (mmHg) | 2700 | 18.90 |
| DBP (mmHg) | 2704 | 18.93 |
| TC (mmol/L) | 2646 | 18.52 |
| TG (mmol/L) | 2675 | 18.72 |
| CREA (μmol/L) | 859 | 6.01 |
| UA (μmol/L) | 860 | 6.02 |
| K (mmol/L) | 610 | 4.27 |
| P (mmol/L) | 2890 | 20.23 |
| Cl (mmol/L) | 611 | 4.28 |
| Na (mmol/L) | 611 | 4.28 |
| Ca (mmol/L) | 2508 | 17.56 |
| NLR | 0 | 0 |
| PLR | 0 | 0 |
| LMR | 0 | 0 |
| PIV | 0 | 0 |
| FIB-4 | 0 | 0 |
| PNI | 0 | 0 |
| FAR | 0 | 0 |
| LDL-C/HDL-C | 0 | 0 |
| NHHR | 0 | 0 |
| CRP/ALB | 0 | 0 |

Abbreviations: **HF**, heart failure; **DM**, diabetes mellitus; **COPD**, chronic obstructive pulmonary disease; **CG**, chronic gastritis; **SBP**, systolic blood pressure; **DBP**, diastolic blood pressure; **TC**, total cholesterol; **TG**, triglycerides; **CREA**, creatinine; **UA**, uric acid; **K**, potassium; **P**, phosphorus; **Cl**, chloride; **Na**, sodium; **Ca**, calcium; **NLR**, neutrophil-to-lymphocyte ratio; **PLR**, platelet-to-lymphocyte ratio; **LMR**, lymphocyte-to-monocyte ratio; **PIV**, pan-immune-inflammation value; **FIB-4**, fibrosis-4 index; **PNI**, prognostic nutritional index; **FAR**, fibrinogen-to-albumin ratio; **LDL-C/HDL-C**, low-density lipoprotein cholesterol/high-density lipoprotein cholesterol; **NHHR**, non-high-density lipoprotein cholesterol to high-density lipoprotein cholesterol ratio; **CRP/ALB**, c-reactive protein to albumin ratio.

**Table S3.** Optimal hyperparameters for the eight ML models

| **Algorithms** | **Hyperparameters** |
| --- | --- |
| LR (logistic regression) | 'random_state': 2026, 'tol': 1e-05, 'penalty': 'l2', 'C': 0.8, 'solver': 'liblinear', 'class_weight': 'balanced' |
| RF (random forest) | 'n_estimators': 300, 'criterion': 'gini', 'max_depth': 6, 'min_samples_split': 5, 'min_samples_leaf': 3, 'max_features': 'sqrt', 'random_state': 42, 'n_jobs': -1, 'class_weight': 'balanced' |
| DT (decision tree) | 'criterion': 'gini', 'max_depth': 5, 'min_samples_split': 5, 'min_samples_leaf': 3, 'random_state': 42, 'class_weight': 'balanced' |
| XGBoost (extreme gradient boosting) | 'subsample': 0.8, 'reg_lambda': 0.2, 'n_estimators': 800, 'min_child_weight': 10, 'max_depth': 5, 'learning_rate': 0.01, 'gamma': 0.2, 'colsample_bytree': 0.6, 'scale_pos_weight': imb_ratio, 'objective': 'binary:logistic', 'eval_metric': 'auc', 'random_state': 42, 'nthread': -1 |
| HistGBDT (histogram-based gradient boosting) | 'max_iter': 300, 'learning_rate': 0.05, 'max_depth': 5, 'l2_regularization': 1.0, 'class_weight': 'balanced', 'random_state': 42 |
| LightGBM (light gradient boosting machine) | 'subsample_freq': 1, 'subsample': 0.8, 'reg_lambda': 1.0, 'reg_alpha': 0.0, 'num_leaves': 31, 'min_child_samples': 20, 'max_depth': 5, 'learning_rate': 0.02, 'colsample_bytree': 0.8, 'random_state': 2025, 'class_weight': 'balanced', 'n_jobs': -1, 'verbose': -1 |
| AdaBoost (adaptive boosting) | 'n_estimators': 150, 'learning_rate': 0.05, 'algorithm': 'SAMME', 'random_state': 42, 'estimator': DecisionTreeClassifier(max_depth=2, min_samples_split=3, min_samples_leaf=4, criterion='gini', random_state=42, class_weight='balanced') |
| NB (naive bayes) | 'var_smoothing': 1e-09 |

**Table S4.** Missing data imputation results for variables with a missing rate below 30% in the training set

| **Variables** | **Number of missing cases** | **Proportion of missing data (%)** | **Before interpolation** | **After interpolation** | ***P* value** |
| --- | --- | --- | --- | --- | --- |
| Pulse rate (beats/min) | 275 | 0.83 | 80.00 (71.00, 90.00) | 80.00 (71.00, 90.00) | 0.984 |
| SBP (mmHg) | 6,111 | 18.33 | 134.00 (122.00, 149.00) | 134.00 (122.00, 149.00) | 0.845 |
| DBP (mmHg) | 6,134 | 18.40 | 79.00 (71.00, 88.00) | 80.00 (71.00, 88.00) | 0.399 |
| TC (mmol/L) | 6,399 | 19.20 | 3.94 (3.25, 4.74) | 3.92 (3.24, 4.70) | 0.061 |
| TG (mmol/L) | 6,471 | 19.41 | 1.21 (0.88, 1.75) | 1.20 (0.87, 1.74) | 0.069 |
| CREA (μmol/L) | 2,095 | 6.29 | 73.00 (59.00, 95.00) | 73.00 (59.00, 95.00) | 0.691 |
| UA (μmol/L) | 2,096 | 6.29 | 339.00 (272.00, 420.00) | 338.00 (272.00, 420.00) | 0.697 |
| K (mmol/L) | 1,538 | 4.61 | 3.83 (3.53, 4.14) | 3.83 (3.54, 4.14) | 0.502 |
| P (mmol/L) | 6,934 | 20.80 | 1.11 (0.97, 1.27) | 1.10 (0.96, 1.26) | 0.002 |
| Cl (mmol/L) | 1,537 | 4.61 | 105.00 (102.00, 108.00) | 105.00 (102.00, 108.00) | 0.779 |
| Na (mmol/L) | 1,537 | 4.61 | 141.00 (138.00, 143.00) | 141.00 (138.00, 143.00) | 0.912 |
| Ca (mmol/L) | 5,991 | 17.97 | 2.24 (2.14, 2.33) | 2.24 (2.14, 2.33) | 0.523 |

Abbreviations: **SBP**, systolic blood pressure; **DBP**, diastolic blood pressure; **TC**, total cholesterol; **TG**, triglycerides; **CREA**, creatinine; **UA**, uric acid; **K**, potassium; **P**, phosphorus; **Cl**, chloride; **Na**, sodium; **Ca**, calcium.

**Table S5.** Missing data imputation results for variables with a missing rate below 30% in the validation set

| **Variables** | **Number of missing cases** | **Proportion of missing data (%)** | **Before interpolation** | **After interpolation** | ***P* value** |
| --- | --- | --- | --- | --- | --- |
| Pulse rate (beats/min) | 130 | 0.91 | 80.00 (71.00, 91.00) | 80.00 (71.00, 91.00) | 0.909 |
| SBP (mmHg) | 2,700 | 18.90 | 134.00 (122.00, 149.00) | 134.00 (122.00, 149.00) | 0.848 |
| DBP (mmHg) | 2,704 | 18.93 | 79.00 (71.00, 88.00) | 80.00 (71.00, 89.00) | 0.243 |
| TC (mmol/L) | 2,646 | 18.52 | 3.94 (3.25, 4.73) | 3.93 (3.25, 4.71) | 0.509 |
| TG (mmol/L) | 2,675 | 18.72 | 1.21 (0.87, 1.76) | 1.20 (0.87, 1.75) | 0.243 |
| CREA (μmol/L) | 859 | 6.01 | 73.00 (59.00, 95.00) | 73.00 (59.00, 95.00) | 0.851 |
| UA (μmol/L) | 860 | 6.02 | 339.50 (272.00, 421.00) | 339.00 (271.00, 421.00) | 0.817 |
| K (mmol/L) | 610 | 4.27 | 3.83 (3.53, 4.14) | 3.83 (3.54, 4.14) | 0.487 |
| P (mmol/L) | 2,890 | 20.23 | 1.10 (0.96, 1.27) | 1.10 (0.95, 1.26) | 0.016 |
| Cl (mmol/L) | 611 | 4.28 | 105.00 (102.00, 108.00) | 105.00 (102.00, 108.00) | 0.684 |
| Na (mmol/L) | 611 | 4.28 | 141.00 (138.00, 143.00) | 141.00 (138.00, 143.00) | 0.975 |
| Ca (mmol/L) | 2,508 | 17.56 | 2.24 (2.14, 2.32) | 2.24 (2.14, 2.32) | 0.951 |

Abbreviations: **SBP**, systolic blood pressure; **DBP**, diastolic blood pressure; **TC**, total cholesterol; **TG**, triglycerides; **CREA**, creatinine; **UA**, uric acid; **K**, potassium; **P**, phosphorus; **Cl**, chloride; **Na**, sodium; **Ca**, calcium.

**Table S6.** Collinearity assessment of the thirteen LASSO‑derived predictors: VIF and tolerance

| **Variables** | **VIF** | **Tolerance** |
| --- | --- | --- |
| Age (years) | 1.195 | 0.837 |
| Smoking history | 1.080 | 0.926 |
| HF | 1.032 | 0.969 |
| DM | 1.067 | 0.937 |
| CG | 1.033 | 0.968 |
| Pulse rate (beats/min) | 1.153 | 0.867 |
| SBP (mmHg) | 1.878 | 0.532 |
| DBP (mmHg) | 1.976 | 0.506 |
| TC (mmol/L) | 1.172 | 0.853 |
| TG (mmol/L) | 1.180 | 0.847 |
| CREA (μmol/L) | 1.189 | 0.841 |
| UA (μmol/L) | 1.196 | 0.836 |
| PLR | 1.248 | 0.801 |
| LMR | 1.378 | 0.726 |
| FAR | 1.017 | 0.983 |
| CRP/ALB | 1.140 | 0.877 |

Abbreviations: **VIF**, variance inflation factor; **HF**, heart failure; **DM**, diabetes mellitus; **CG**, chronic gastritis; **SBP**, systolic blood pressure; **DBP**, diastolic blood pressure; **TC**, total cholesterol; **TG**, triglycerides; **CREA**, creatinine; **UA**, uric acid; **PLR**, platelet-to-lymphocyte ratio; **LMR**, lymphocyte-to-monocyte ratio; **FAR**, fibrinogen-to-albumin ratio; **CRP/ALB**, c-reactive protein to albumin ratio.

**Table S7.** Per‑class performance metrics of eight ML models on the validation set

| **Model** | **AF class (positive)** | | | **Non‑AF class (negative)** | | |
| --- | --- | --- | --- | --- | --- | --- |
|  | **Precision** | **Recall** | **F1‑score** | **Precision** | **Recall** | **F1‑score** |
| XGBoost | 0.309 | 0.718 | 0.432 | 0.946 | 0.756 | 0.840 |
| GBDT | 0.301 | 0.721 | 0.425 | 0.946 | 0.745 | 0.833 |
| LightGBM | 0.282 | 0.735 | 0.407 | 0.946 | 0.714 | 0.814 |
| RF | 0.280 | 0.729 | 0.404 | 0.945 | 0.714 | 0.813 |
| AdaBoost | 0.192 | 0.817 | 0.312 | 0.945 | 0.477 | 0.634 |
| LR | 0.275 | 0.736 | 0.401 | 0.946 | 0.704 | 0.807 |
| DT | 0.275 | 0.637 | 0.384 | 0.931 | 0.744 | 0.827 |
| NB | 0.255 | 0.737 | 0.379 | 0.944 | 0.672 | 0.785 |

Abbreviations: **XGBoost**, extreme Gradient Boosting; **GBDT**, gradient boosting decision tree; **LightGBM**, light gradient boosting machine; **RF**, random forest; **AdaBoost**, adaptive boosting; **LR**, logistic regression; **DT**, decision tree; **NB**, naive Bayes.

**Table S8.** Fold‑wise AUCs of eight ML models in stratified 5‑fold cross‑validation

| **Model** | **Fold 1** | **Fold 2** | **Fold 3** | **Fold 4** | **Fold 5** | **Mean AUC** | **SD** |
| --- | --- | --- | --- | --- | --- | --- | --- |
| XGBoost | 0.815 | 0.811 | 0.818 | 0.811 | 0.804 | 0.812 | 0.005 |
| GBDT | 0.811 | 0.806 | 0.814 | 0.809 | 0.802 | 0.809 | 0.005 |
| LightGBM | 0.805 | 0.798 | 0.806 | 0.798 | 0.792 | 0.800 | 0.006 |
| RF | 0.804 | 0.792 | 0.800 | 0.792 | 0.785 | 0.795 | 0.007 |
| AdaBoost | 0.711 | 0.692 | 0.700 | 0.692 | 0.701 | 0.699 | 0.008 |
| LR | 0.798 | 0.791 | 0.799 | 0.797 | 0.788 | 0.795 | 0.005 |
| DT | 0.765 | 0.753 | 0.758 | 0.753 | 0.754 | 0.757 | 0.005 |
| NB | 0.784 | 0.764 | 0.78 | 0.775 | 0.762 | 0.773 | 0.010 |

Abbreviations: **AUC**, area under the ROC curve; **SD**, standard deviation; **XGBoost**, extreme Gradient Boosting; **GBDT**, gradient boosting decision tree; **LightGBM**, light gradient boosting machine; **RF**, random forest; **AdaBoost**, adaptive boosting; **LR**, logistic regression; **DT**, decision tree; **NB**, naive Bayes.

**Table S9.** Mean absolute SHAP values of the 16 LASSO‑selected predictors in the XGBoost model

| **Rank** | **Feature** | **Mean absolute SHAP value** |
| --- | --- | --- |
| 1 | Pulse rate (beats/min) | 1.036 |
| 2 | TC (mmol/L) | 0.784 |
| 3 | SBP (mmHg) | 0.598 |
| 4 | CREA (μmol/L) | 0.506 |
| 5 | TG (mmol/L) | 0.503 |
| 6 | DBP (mmHg) | 0.465 |
| 7 | HF | 0.465 |
| 8 | FAR | 0.365 |
| 9 | PLR | 0.302 |
| 10 | LMR | 0.291 |
| 11 | Age (years) | 0.280 |
| 12 | UA (μmol/L) | 0.253 |
| 13 | CRP/ALB | 0.071 |
| 14 | DM | 0.039 |
| 15 | CG | 0.019 |
| 16 | Smoking history | 0.017 |

Abbreviations: **SHAP**, Shapley additive explanations; **TC**, total cholesterol; **SBP**, systolic blood pressure; **CREA**, creatinine; **TG**, triglycerides; **DBP**, diastolic blood pressure; **HF**, heart failure; **FAR**, fibrinogen-to-albumin ratio; **PLR**, platelet-to-lymphocyte ratio; **LMR**, lymphocyte-to-monocyte ratio; **UA**, uric acid; **CRP/ALB**, c-reactive protein to albumin ratio; **DM**, diabetes mellitus; **CG**, chronic gastritis.

**Table S10.** Ablation analysis results on the validation set

| **Ablation type** | **Configuration** | **Validation AUC** | **Δ vs. full model** |
| --- | --- | --- | --- |
| Full model | All 16 predictors + RF imputation + GridSearch | 0.813 | — |
| Feature ablation | Remove pulse rate | 0.796 | −0.017 |
|  | Remove TC | 0.801 | −0.012 |
|  | Remove SBP | 0.803 | −0.010 |
|  | Remove CREA | 0.805 | −0.008 |
|  | Remove TG | 0.809 | −0.004 |
|  | Remove all top‑5 | 0.784 | −0.029 |
| Preprocessing ablation | Mean/median imputation | 0.792 | −0.021 |
|  | Complete‑case analysis | 0.801 | −0.012 |
| Optimizer ablation | Default hyperparameters | 0.787 | −0.026 |

Abbreviations: **AUC**, area under the ROC curve; **TC**, total cholesterol; **SBP**, systolic blood pressure; **CREA**, creatinine; **TG**, triglycerides

**
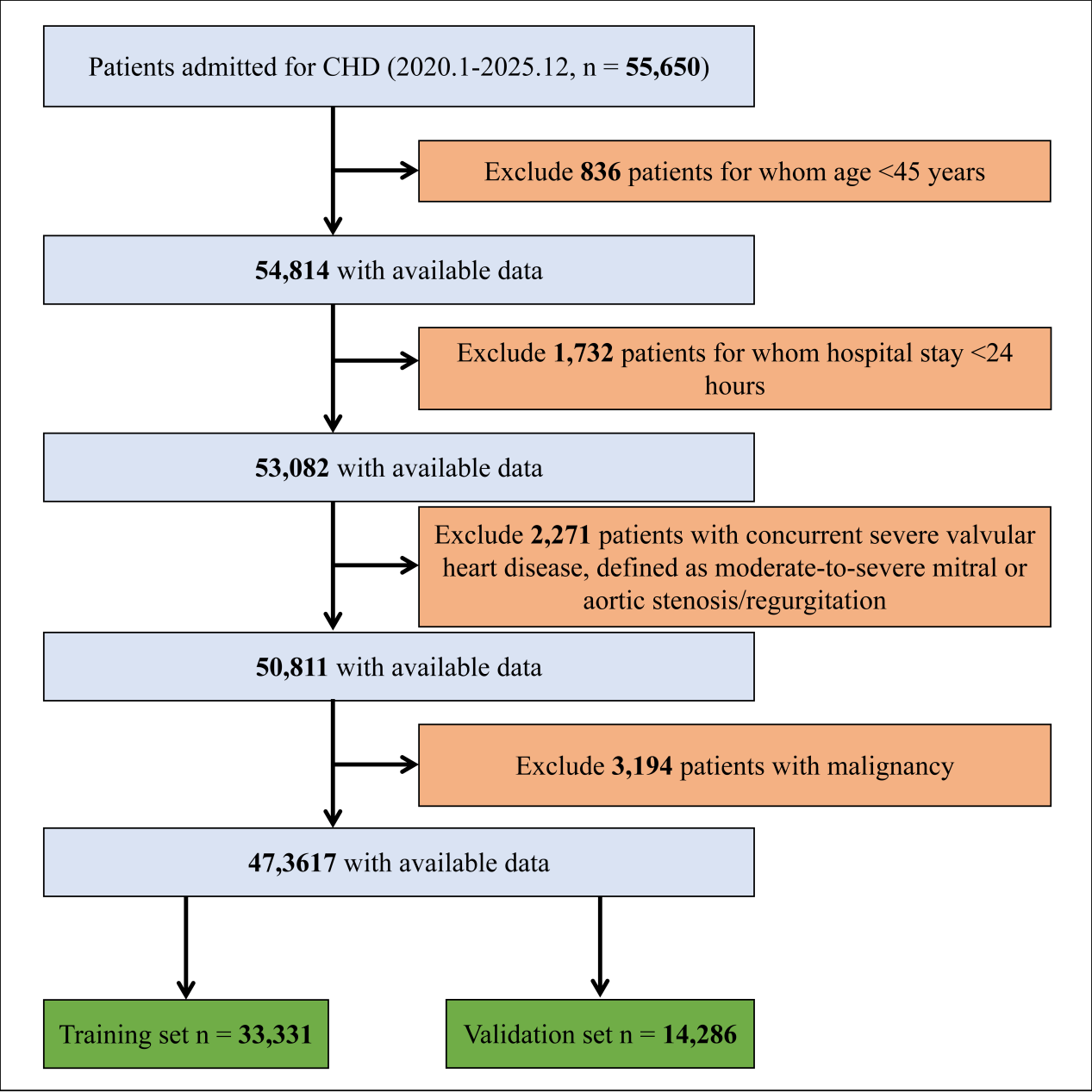
**

**Figure S1.** Patient selection and data partitioning workflow


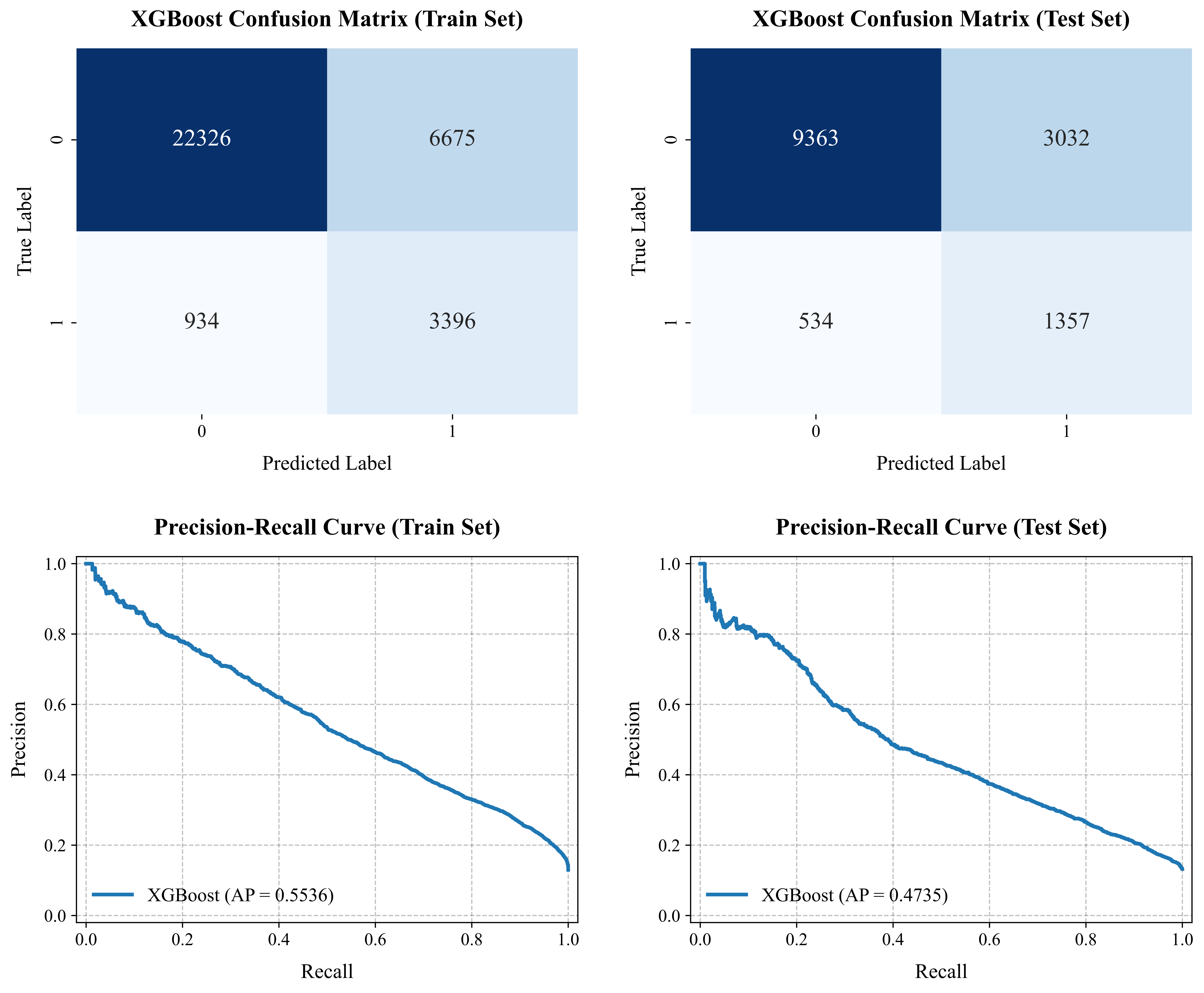


**Figure S2.** Confusion matrices and Precision‑Recall (PR) curves for the XGBoost model on the training and validation sets
